# Cell surface expression of GRP78 and CXCR4 is associated with childhood high-risk acute lymphoblastic leukemia at diagnostics

**DOI:** 10.1038/s41598-022-05857-w

**Published:** 2022-02-11

**Authors:** Tania Angeles-Floriano, Guadalupe Rivera-Torruco, Paulina García-Maldonado, Esmeralda Juárez, Yolanda Gonzalez, Israel Parra-Ortega, Armando Vilchis-Ordoñez, Briceida Lopez-Martinez, Lourdes Arriaga-Pizano, Dario Orozco-Ruíz, José Refugio Torres-Nava, Paula Licona-Limón, Francisco López-Sosa, Alhelí Bremer, Lourdes Alvarez-Arellano, Ricardo Valle-Rios

**Affiliations:** 1 Unidad Universitaria de Investigación, División de Investigación, Facultad de Medicina, UNAM-Hospital Infantil de México Federico Gómez, Universidad 3000, CP 04510 Mexico City, Mexico; 2 Programa de Maestría y Doctorado en Ciencias Médicas Odontológicas y de la Salud, Universidad Nacional Autónoma de México (UNAM), Mexico City, Mexico; 3 Unidad de Investigación en Inmunología y Proteómica, Hospital Infantil de México Federico Gómez, Mexico City, Mexico; 4 Departamento de Fisiología y Neurociencias, Centro de Investigación y de Estudios Avanzados (CINVESTAV), Mexico City, Mexico; 5 Departamento de Investigación en Microbiología, Instituto Nacional de Enfermedades Respiratorias Ismael Cosío Villegas, Mexico City, Mexico; 6 Subdirección de Diagnóstico clínico y Departamento de Laboratorio Clínico, Hospital Infantil de México Federico Gómez, Mexico City, Mexico; 7 Unidad de Investigación Médica en Inmunoquímica, CMN Siglo XXI, IMSS, Mexico City, Mexico; 8 Hospital Pediátrico Moctezuma, Mexico City, Mexico; 9 Departamento de Biología Celular y del Desarrollo, Instituto de Fisiología Celular, Universidad Nacional Autónoma de México (UNAM), Mexico City, Mexico; 10 Departamento de Ortopedia, Hospital Infantil de México Federico Gómez, Mexico City, Mexico; 11 CONACYT-Hospital Infantil de México Federico Gómez, Mexico City, Mexico

**Keywords:** Biomarkers, Oncology

## Abstract

Acute lymphocytic leukemia is the most common type of cancer in pediatric individuals. Glucose regulated protein (GRP78) is an endoplasmic reticulum chaperone that facilitates the folding and assembly of proteins and regulates the unfolded protein response pathway. GRP78 has a role in survival of cancer and metastasis and cell-surface associated GRP78 (sGRP78) is expressed on cancer cells but not in normal cells. Here, we explored the presence of sGRP78 in pediatric B-ALL at diagnosis and investigated the correlation with bona fide markers of leukemia. By using a combination of flow cytometry and high multidimensional analysis, we found a distinctive cluster containing high levels of sGRP78, CD10, CD19, and CXCR4 in bone marrow samples obtained from High-risk leukemia patients, which was absent in the compartment of Standard-risk leukemia. We confirmed that sGRP78^+^CXCR4^+^ blood-derived cells were more frequent in High-risk leukemia patients. Finally, we analyzed the dissemination capacity of sGRP78 leukemia cells in a model of xenotransplantation. sGRP78^+^ cells emigrated to the bone marrow and lymph nodes, maintaining the expression of CXCR4. Testing the presence of sGRP78 and CXCR4 together with conventional markers may help to achieve a better categorization of High and Standard-risk pediatric leukemia at diagnosis.

## Introduction

The 78-kDa immunoglobulin heavy chain binding protein BIP/HSPA5, also known as glucose regulated protein (GRP78) is an endoplasmic reticulum (ER) resident molecular chaperone that belongs to the Hsp70 family of heat shock proteins and it is a component of the unfolded protein response (UPR) pathway that plays an important role in ER homeostasis^1–3^. In normal conditions, GRP78 interacts with PKR-like ER kinase (PERK), inositol-requiring enzyme 1 (IRE1), and activating transcription factor 6 (ATF6). On accumulation of defective proteins in ER, GRP78 dissociates from these sensors and releases them to initiate the UPR cascade^4^.

The activation of the UPR cascade seems to be important for integrity maintenance of hematopoietic stem cells (HSCs) by preventing the propagation of damaged cells, reducing malignancy risk^5^. However, UPR cascade also represents a selective advantage to preleukemic stem cells, promoting their clonal expansion^6^.

As a master regulator of ER stress, GRP78 has been proposed to be important for stem cell survival, since GRP78 deficient mice showed enhanced cell death leading to a loss of the HSC compartment^7^.

Cell surface localization of GRP78 (sGRP78) was first reported in 1997 and recent studies have shown that GRP78 can be translocated to different cell compartments^8^. Importantly, sGRP78 works as a multifunctional receptor at the cell surface on tumor cells, where it triggers different cellular responses associated to proliferation, invasion, apoptosis, inflammation, and immunity^8–12^. Furthermore, sGRP78 has been observed in a variety of tumors including glioblastoma, prostate, breast, lung, gastric, colon and leukemia^13–18^.

GRP78 haploinsufficiency potently suppresses leukemogenesis and AKT/mTOR signaling in PTEN null bone marrow cells and the GRP78 level expression may alter the sensitivity of human leukemic cells to Arabinoside-induced apoptosis. Furthermore, the emerging association of elevated GRP78 expression in leukemic blasts of adult patients suggests that GRP78 may be important in the field of leukemia research^15, 19–21^. However, information about the presence of sGRP78 in childhood leukemia is scarce. Therefore, we aimed to evaluate sGRP78 in pediatric patients with B-lineage acute lymphoblastic leukemia (B-ALL) at diagnosis and investigate its association with stem cell markers (CD34 and CD38), lineage markers (CD10 and CD19). Also, sGRP78 expression was compared with CXCR4 expression, a well-known biomarker associated with extramedullary organ infiltration in childhood leukemia^22^.

## Materials and methods

### Human samples

Patients newly diagnosed with B-ALL were recruited from the Hospital Infantil de México Federico Gómez and Hospital Pediátrico de Moctezuma in México City. Bone marrow (BM) samples were collected according to International and Institutional Guidelines from children and adolescents younger than 18 years and diagnosed with ALL before any treatment. Control samples were obtained from children undergoing orthopedic surgery without diagnosis of oncology or hematology diseases or solid tumors, according with international and institutional guidelines. National Cancer Institute (NCI)-Rome guidelines were used for risk classification. High: WBC ≥ 50 × 10^9^/L or ≥ 10 years; standard: WBC < 50 × 10^9^/L and age 1 < 10 years.

A total of 43 newly diagnosed patients with B-ALL and 13 controls were used in this study (Supplementary Fig. 1). 43 B-ALL samples from BM and 4 BM controls were used for multiparametric flow cytometry. 9 blood control samples and 26 B-ALL blood samples from newly diagnosed patients with B-ALL were also analyzed. (Supplementary Fig. 1)

5 Standard-risk bone marrow B-ALL samples and 6 High-risk samples (from the described 43 samples) were used for flow cytometry and FlowSOM clustering analysis. Finally, 3 High risk B-ALL samples of bone marrow were used in xenotransplant assays (from the described 43 samples).

### Ethics statement

Only remnant samples were collected, and all protocols and investigations of our study were approved by the ethics committees of Hospital Infantil de México Federico Gómez (HIM-2016-023) and Hospital Pediátrico de Moctezuma in México City (2016-01-3385). Patients and their parents signed the informed consent.

The study was conducted in accordance to Declaration of Helsinki.

### Cell lines and culture conditions

SUP-B15, RS4;11, and Reh ALL cell lines were purchased from American Type Culture Collection (ATCC, Rockville, MD, USA). The cells were cultured according to ATCC guidelines and maintained in 5% CO_2_ incubator at 37 °C. Freshly harvested cells were counted and resuspended in PBS with 2% FBS to follow the flow cytometry staining.

### Isolation of mononuclear cells

Mononuclear cells (MNC) were isolated from BM and peripheral blood by using Ficoll-High Paque separation media. Briefly, a centrifuge tube with a volume of 1 to 2 mL of Ficoll-High Paque separation media was used to carefully place a mixture of BM or blood with PBS1x (1:1 volume ratio) and then centrifuged at 800×*g* for 30 min, at RT with the brake off. Harvested cells were washed with 5 mL of PBS1x twice. Cell viability was evaluated using trypan blue dye exclusion test.

### Multiparameter flow cytometry

1–2 × 10^6^ cells were placed in 200 uL of PBS1X with 2% FBS on ice and antibodies were added, mixed, and incubated for 30 min. Then, cells were washed with PBS1X and centrifuged at 800×*g* for 5 min. Finally, samples were acquired in a CytoFLEX LX cytometer (Beckman Coulter). Analysis was performed using CytExpert software. The following anti human antibodies were used: AF488 anti-GRP78 (Clone: C38), AF700 anti-CD45 (Clone: HI30), APC anti-CD34 (Clone: 561), PE/Cy7 anti-CD38 (Clone: HB-7), PerCP/Cy5.5 anti-CD19 (Clone: HIN19), PE/Dazzle anti-CD10 (Clone: HI10a), Brilliant Violet 421 anti-CD184 (clone: 12G5) and the Zombie Aqua™ Fixable Viability Kit was used according to the manufacture (Biolegend).

### FlowSOM clustering and tSNE representation

For the analysis, after Flow Cytometry acquisition, possible debris and doublets events were excluded, LiveCD45^+^ cells from each sample were gated and 40,000 events were exported using CytExpert2.0. Pre-processed and compensated FCS files were loaded and analyzed into R Studio with the packages flowCore, flowVix, flowSOM and CATALYST^23, 24^. tSNE is based on the arcsinh-transformed expression of Live CD45^+^ cells stained with the LLA lineage markers: CD38, CD19, CD10, CD34, CXCR4, and GRP78. Clustering was run with no PCA, 2000 cells were randomly selected from each sample and the represented dimensional reduction was made with tSNE.

### Mice

Recipient BALB/c nude immunodeficient mice were obtained from Instituto Nacional de Nutrición Salvador Zubirán (INCMNSZ) and maintained in a specific pathogen-free environment throughout the experiments. All animal-related experimental protocols were approved by the animal research committee at Hospital Infantil de México Federico Gómez (HIM-2019-031). All methods are reported in accordance with ARRIVE guidelines. We confirm that all methods were performed in accordance with the relevant guidelines and regulations.

### Xenotransplant assay

To evaluate the dissemination capacity of sGRP78 cells, 250,000 to 500,000 sGRP78 sorted cells using *FACS Aria IIu*, were embedded in 100 uL of RPMI and 100 uL of Matrigel and injected subcutaneously into the right flank of each mouse (n = 3). Mice were examined daily and 3 weeks upon xenotransplantation, bone marrow and inguinal lymph node cells were recovered and analyzed by flow cytometry. Due to anti-GRP78 antibody cross-reacted against mouse cells, we selected human cells by using anti human CD45 antibody (HI30), then CD45^+^ human cells were gated for sGRP78, CD34 (581) and CXCR4 (12G5) detection. Both anti CD34 and anti CXCR4 recognized only human cells.

## Results

### High proportion of bone marrow lymphoblastic leukemia cells express sGRP78

**Figure 1:**
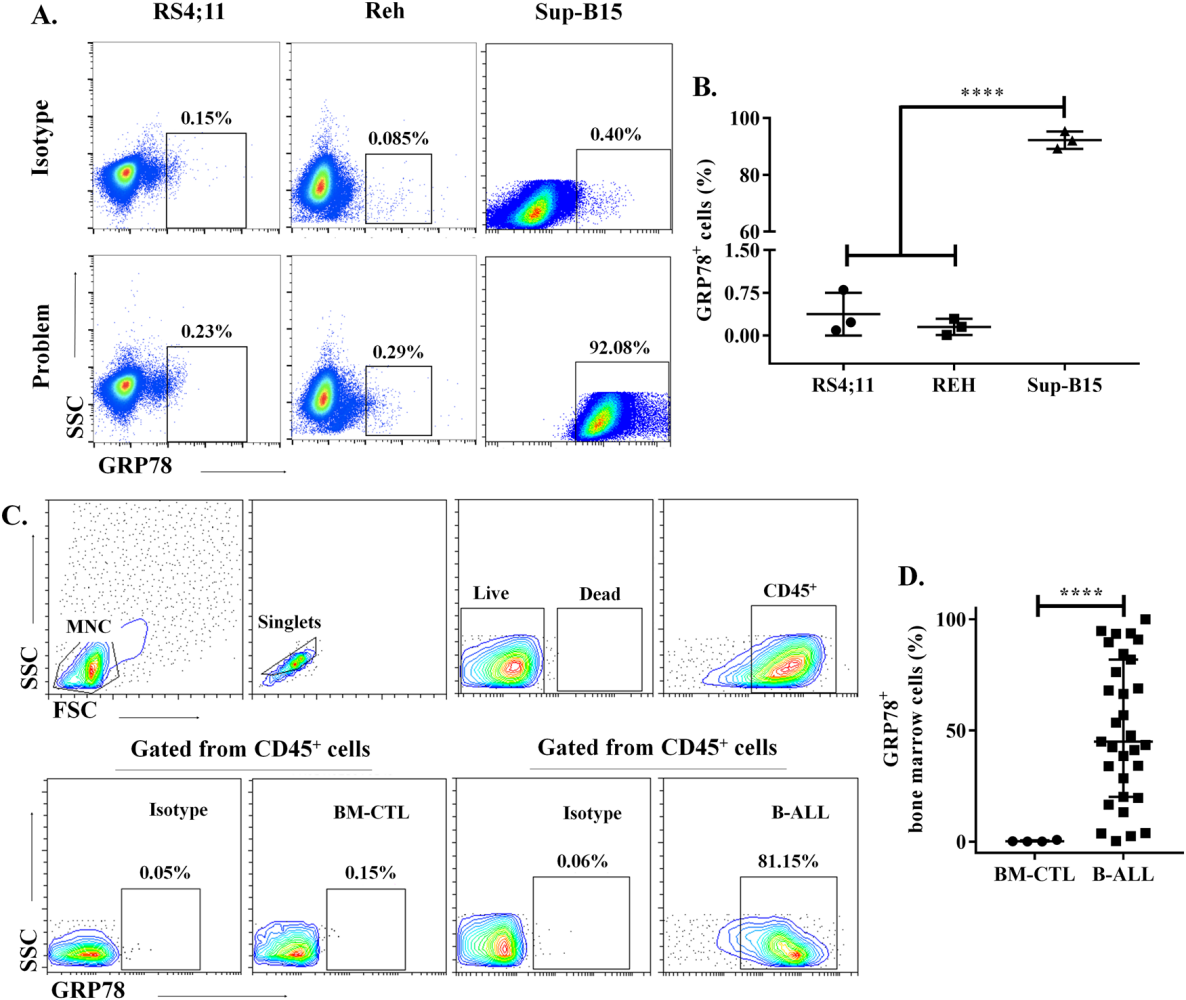
sGRP78 expression on bone marrow cells of pediatric individuals with newly diagnosed B-ALL. (**A**) Acute lymphocytic leukemia cell lines were stained with anti-GRP78 antibody and representative experiments of each cell line are depicted. (**B**) Statistical representation of cell surface GRP78^+^ cells in leukemic cells lines is shown. Data are the mean plus SEM of n = 3. Statistical analysis was performed by One-way ANOVA, p < 0.0001. (**C**) Gating strategy for sGRP78^+^ cells analysis from bone marrow derived cells of pediatric individuals with B-ALL and controls (BM-CTL). (**D**) Statistical representation of cell surface GRP78^+^ cells in BM-CTL (n = 4) and B-ALL (n = 43). Statistical analysis was performed by two-tailed Mann–Whitney U test, *p < 0.05, **p < 0.01.

We assessed the expression of sGRP78 by flow cytometry in ALL cell lines (Fig. 1A). We found that around 90% of Sup-B15 cells, expressed sGRP78. Conversely, less than 1% of Reh and RS4;11 cell lines expressed sGRP78 (Fig. 1B). Thus, the expression of sGRP78 may be different according to the leukemic cell line tested. Then we decided to analyze sGRP78 expression in pediatric acute leukemia by flow cytometry analysis for GRP78 detection on pediatric bone marrow samples with B cell acute leukemia at diagnosis, classified in Standard and High-risk leukemia (Fig. 1C, Table 1). We observed that a high proportion of live single CD45^+^ leukemia cells expressed sGRP78, interestingly healthy cells barely expressed sGRP78 (Fig. 1D).

**Table 1:** Patients and characteristics.

ID	Age (years)	Sex	Leucocytes 10^3^/uL	Blasts %	Translocations	Risk
1	2	F	4.93	80	–	Standard
2	5	F	1.6	83	–	Standard
3	7	F	114	87	–	High
4	8	F	6.59	92	–	Standard
5	4	M	70.15	89	–	High
6	14	M	1.48	55	–	High
7	12	M	1.059	100	–	High
8	6	F	3.3	83	–	Standard
9	5	M	155	97	–	High
10	12	F	3.87	80	–	High
11	6	M	1.55	60	–	Standard
12	5	M	35	97	Neg	Standard
13	6	M	17.5	84	–	Standard
14	2	F	2.1	81	–	Standard
15	15	M	61.2	92	–	High
16	2	F	79.88	97	–	High
17	3	F	1.520	60	–	Standard
18	10	M	269	100	–	High
19	4	F	73	98	t(4;11)	High
20	1a	F	3.9	85	t(4;11)	Standard
21	7	M	15	60	Neg	Standard
22	6	M	540.4	77	–	High
23	5	F	37.39	97	–	Standard
24	4	M	31	89.3	–	Standard
25	12	M	41	95	Neg	High
26	17	F	8	64	–	High
27	16	F	12.5	83	–	High
28	3	F	98	56	t(1;19)	High
29	1	F	29	–	–	Standard
30	7	F	25.1	–	–	Standard
31	15	M	287.7	94	Neg	High
32	5	F	71.4	98	t(1;19)	High
33	10	F	–	–	Neg	High
34	6	F	52.4	78	–	High
35	5	F	90.8	99.5	Neg	High
36	4	M	8.6	96.5	–	Standard
37	15	F	176	97	t(12;21)	High
38	4	M	25.7	90	–	Habitual
39	2	M	47.92	93	–	Habitual
40	12	F	185.2	83	Neg	High
41	13	F	36.2	96.5	t(16;21)	High
42	10	F	184.5	–	–	High
43	5	M	60.4	6	–	High

Risk stratification is shown.

### sGRP78^+^ cells are associated with CD34, CD38, CD10 and CXCR4 expression in the high-risk group of B-ALL patients

**Figure 2:**
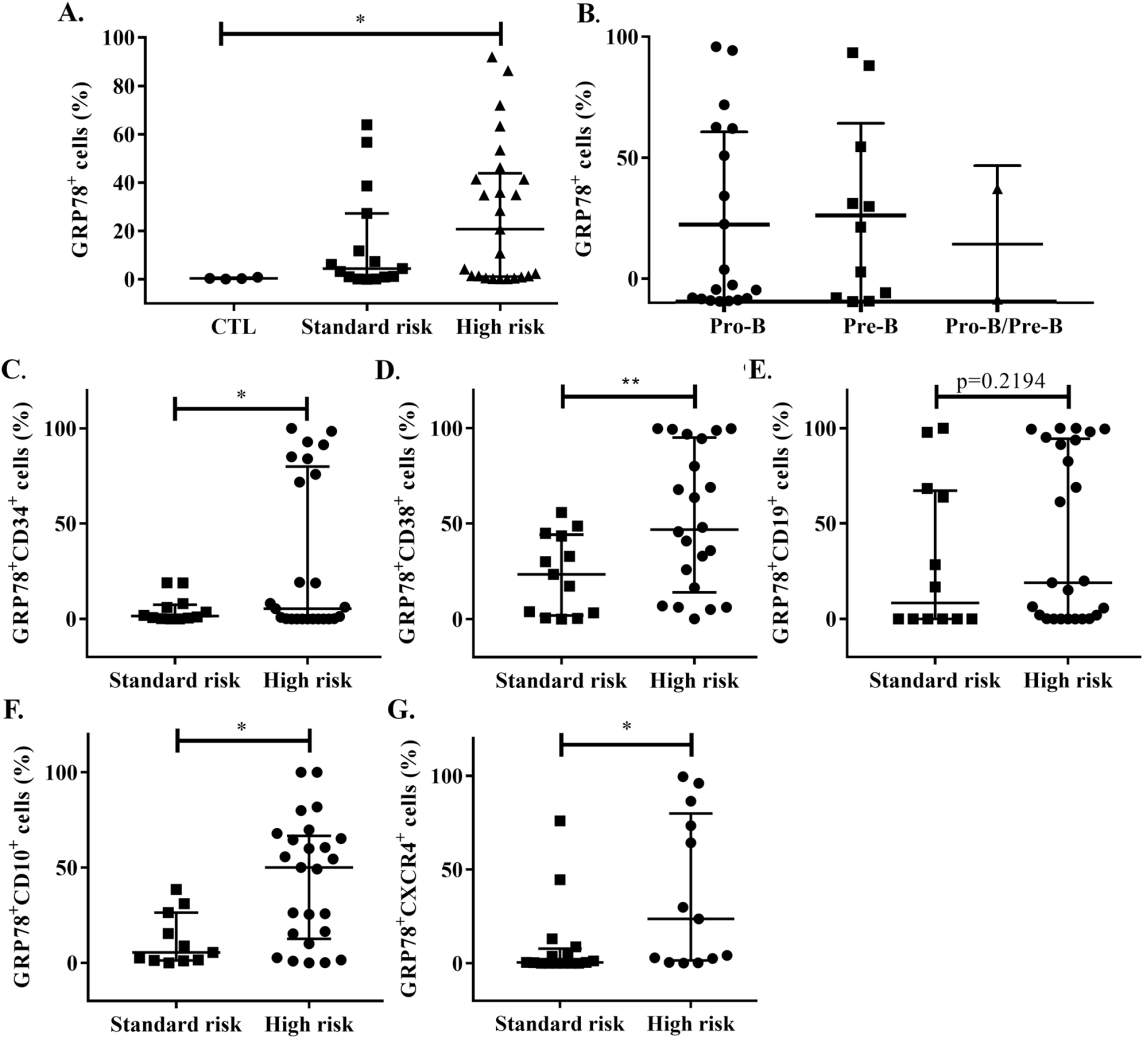
Progenitor and migration markers are associated with GRP78^+^ cells in High-risk individuals with B-ALL. (**A,B**) Patients were classified by Standard risk and High risk and according to the stage of B-cell development markers, and the percentage of sGRP78^+^ cells was assessed. Statistical analysis was performed by two-tailed Mann–Whitney U test, *p < 0.01, **p < 0.05. (**C–G**) CD34, CD38, CD10, CD19 and CXCR4 were measured on GRP78^+^ cells by flow cytometry and the percentages are depicted for both high and standard-risk patients. Statistical analysis was performed by Mann–Whitney U test, *p < 0.01, **p < 0.05, ***p < 0.001.

Since we observed a high proportion of sGRP78^+^ cells in bone marrow samples from children with newly diagnosed B acute lymphoblastic leukemia, we wanted to evaluate the association of sGRP78^+^ cells with other clinical criteria for leukemia classification. Analysis of the proportion of sGRP78^+^ cells according to the risk group of leukemia showed no differences between them (Fig. 2A), also we found no differences between the proportion of sGRP78^+^ cells with the stages of B cell development (ProB, PreB and ProB/PreB cells) (Fig. 2B). However, since initial analysis of leukemia cells by ImageStream showed coexpression of CD34 and sGRP78 in single cells derived from High and Standard-risk patients (Supplementary Fig. 2), we decided to analyze a set of classical surface antigens on leukemia cells. We observed a higher proportion of sGRP78^+^ cells coexpressing CD34, CD38 or CD10 in the High-risk group compared to the Standard-risk group, but we found no differences in the proportion of sGRP78^+^ expressing CD19 (Fig. 2C–F).

sGRP78 has been associated with tumor initiating cells (TICs) in breast cancer which also express elevated levels of the chemokine receptor CXCR4^22, 25^. Since B-ALL express CXCR4 and it has been associated to extramedullary organ infiltration correlating with unfavorable outcome^26^, thus, we decide to evaluate CXCR4 in sGRP78^+^ B-ALL samples. We observed that the percentage of sGRP78^+^CXCR4^+^ cells was associated with the High-risk group of B-ALL patients (Fig. 2G).

Interestingly, though the proportions of sGRP78^+^ cells coexpressing CD34, CD38, CD10 or CXCR4 were significantly higher in High-risk leukemia population, these samples showed the presence of a subgroup of double positive cells rather than a general increase of the positivity to the analyzed markers (Fig. 2C,D,F,G).

### Differential distribution of sGRP78 B-ALL cells in standard vs high-risk B-ALL patients showed by high dimensional analysis

**Figure 3:**
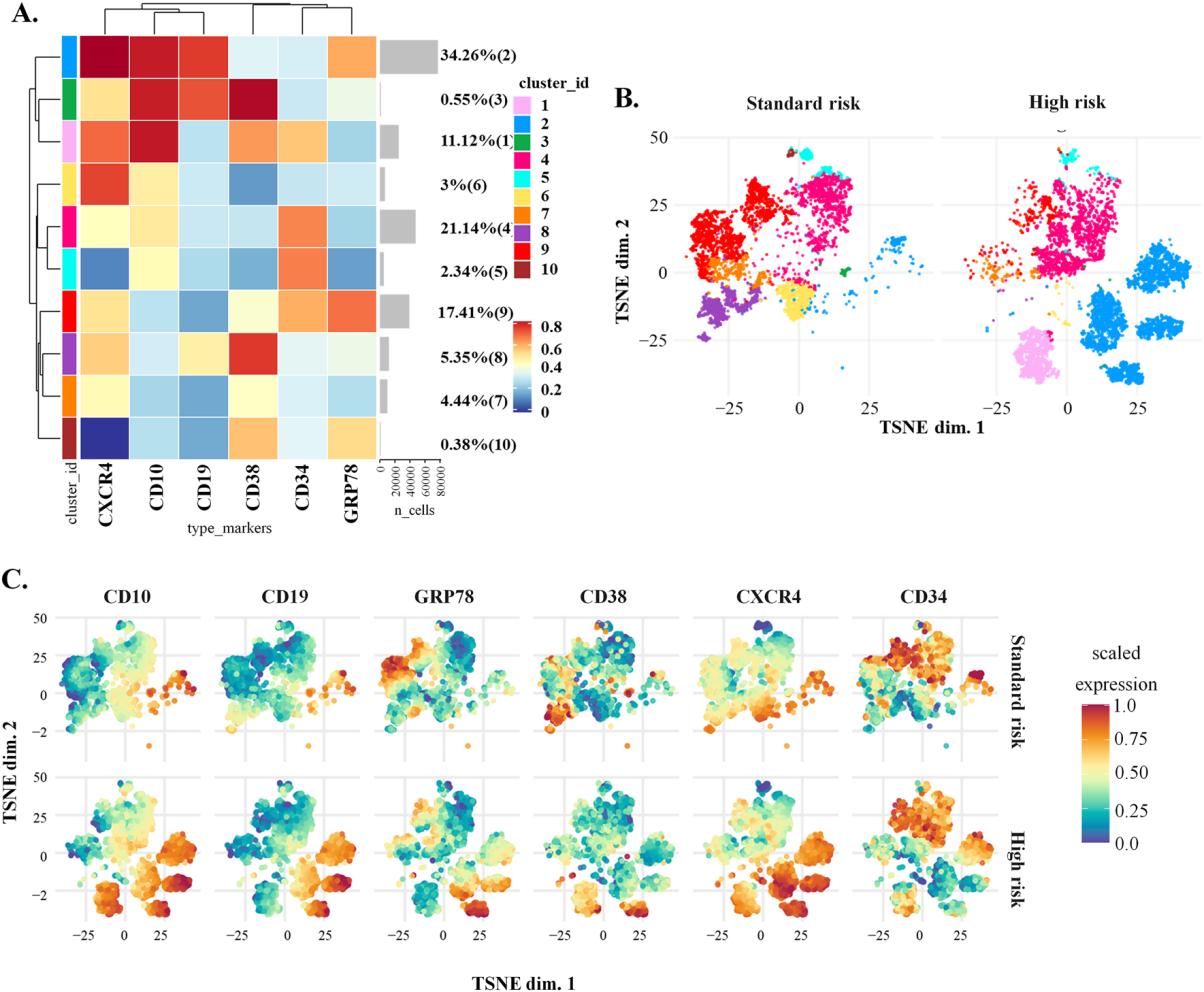
Dimensional reduction of flow cytometry data from Standard and High-risk LLA patients. (**A**) Cluster organization and heatmap of the median marker intensities of the lineage markers and sGRP78. (**B**) TSNE based on the 10 clusters obtained by FlowSOM comparing LLA Standard-risk group (Standard-risk) against LLA High-risk group (High-risk). (**C**) TSNE showed the expression level of each marker separated by group risk. Heat maps were built by using Cytometry dATa anALYSis Tools (CATALYST). https://bioconductor.org/packages/3.10/bioc/html/CATALYST.html.

Since conventional flow cytometry evaluation showed significant differences between leukemia cells derived from High and Standard-risk patients when two surface markers were analyzed (sGRP78 in combination with CD34, CD38, CD10 or CXCR4). We wondered whether the analysis of the whole stained markers may highlight the presence of a particular sGRP78^+^ subset of leukemia cells. Therefore, we examined samples from five Standard-risk patients and six High-risk patients using the t-distributed stochastic neighbor embedding (tSNE). We generated a clustered organization of the samples in terms of the expression of CD34, CD38, CD10, CD19 and CXCR4. Ten clusters were generated and two of them showed the highest levels of sGRP78, cluster 2 and cluster 9, representing 34.26% and 17.41% of the whole analyzed cells respectively (Fig. 3A). Since cluster 2 was the most prominent of the 10 generated clusters, we analyzed the sample distribution and found that this cluster was associated with the High-risk group (Fig. 3B). In the other hand, cluster 9 was associated to Standard-risk samples (Fig. 3B). The distribution of the individual markers along the samples confirmed that sGRP78, CXCR4, CD10 and CD19 were enriched in the cluster 2 compared to cluster 9. In the other hand, CD34 and sGRP78 were enriched in the cluster 9, with low levels of CXCR4 (Fig. 3C). Moreover, three distinct clusters showed high levels of CD38 and low expression of CD34 (Clusters 3, 8 and 10), two of them expressed little or none sGRP78 (Cluster 3 and 8) and were only observed in the Standard risk group (Fig. 3C).

### Circulating lymphoblastic leukemia cells express sGRP78 and CXCR4

**Figure 4:**
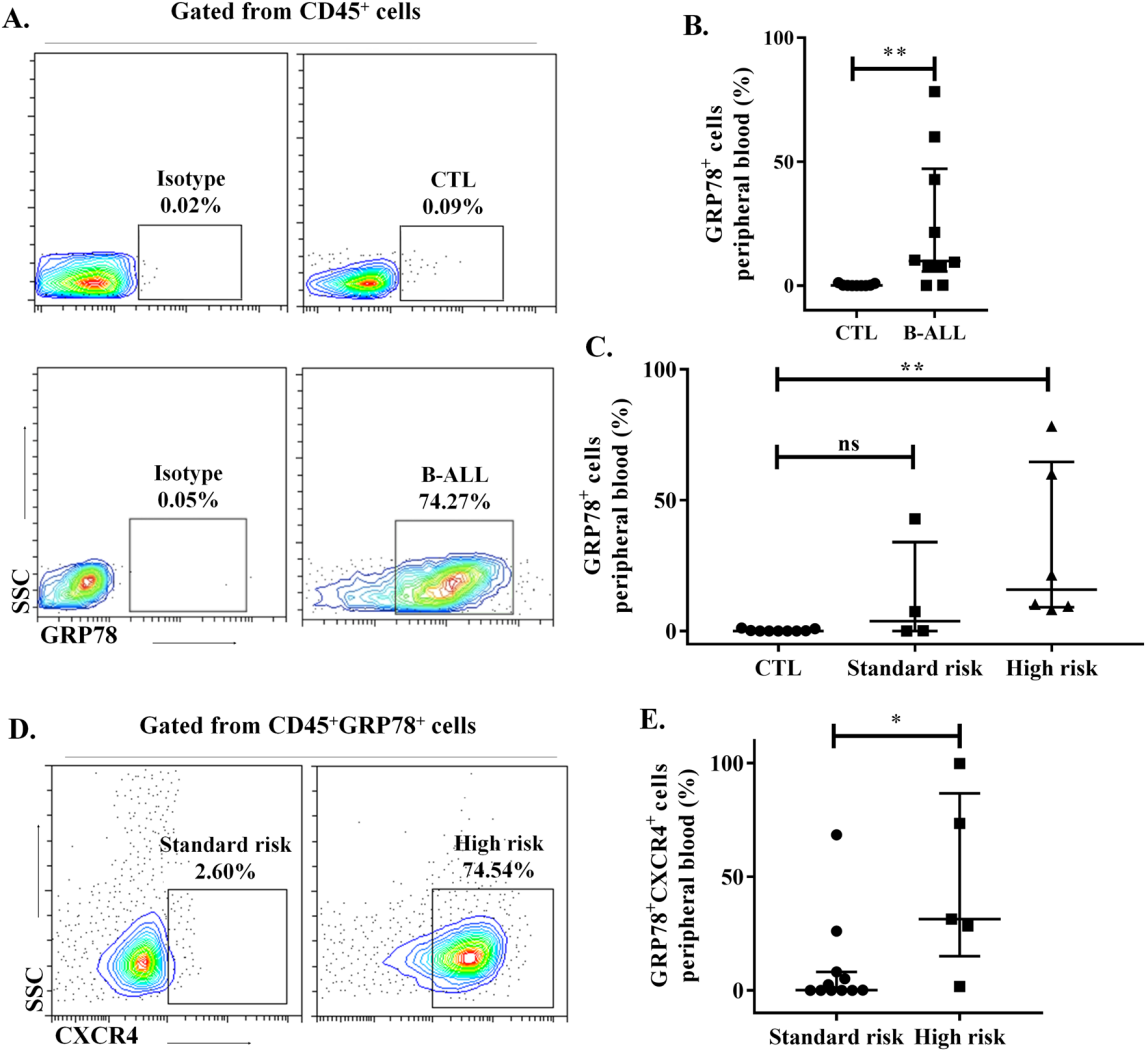
sGRP78^+^ CXCR4^+^ cells are present in peripheral blood of pediatric individuals with newly diagnosed B-ALL. (**A**) Live cells from peripheral blood of B-ALL patients and controls (CTL) were stained with anti-CD45 and anti-GRP78. (**B**) Statistical representation of GRP78^+^ cells in B-ALL patients and controls. (**C**) Percentages of GRP78^+^ cells in high and standard risk are depicted. (**D**) Live cells from peripheral blood were stained with anti-CD45, anti-GRP78 and anti-CXCR4. The plots of a Standard risk patient (left) and High risk patient (right) are depicted. (**E**) Percentages of sGRP78^+^ CXCR4^+^ cells in high and standard risk in peripheral blood. Data are the medians plus ranks. Statistical analysis was performed by two-tailed Mann–Whitney U test, **p < 0.01, ***p < 0.001.

Since leukemia cells have the capacity to emigrate from bone marrow to peripheral tissues, we wondered whether circulating leukemia cells also express sGRP78. We analyzed blood samples from pediatric patients with ALL at diagnostics and compared with samples from healthy donors and observed the presence of CD45^+^GRP78^+^ cells (Fig. 4A,B). Furthermore, cells from patients classified with High-risk leukemia had a higher proportion of sGRP78^+^ cells (Fig. 4C). Similarly, we also observed that High-risk leukemia patients had a higher proportion of sGRP78^+^ CXCR4^+^ cells (Fig. 4D,E). Thus, sGRP78^+^ leukemia cells can be detected in blood and together with the expression of CXCR4.

### Analysis of sGRP78^+^ leukemia cells in a xenotransplantation model

**Figure 5:**
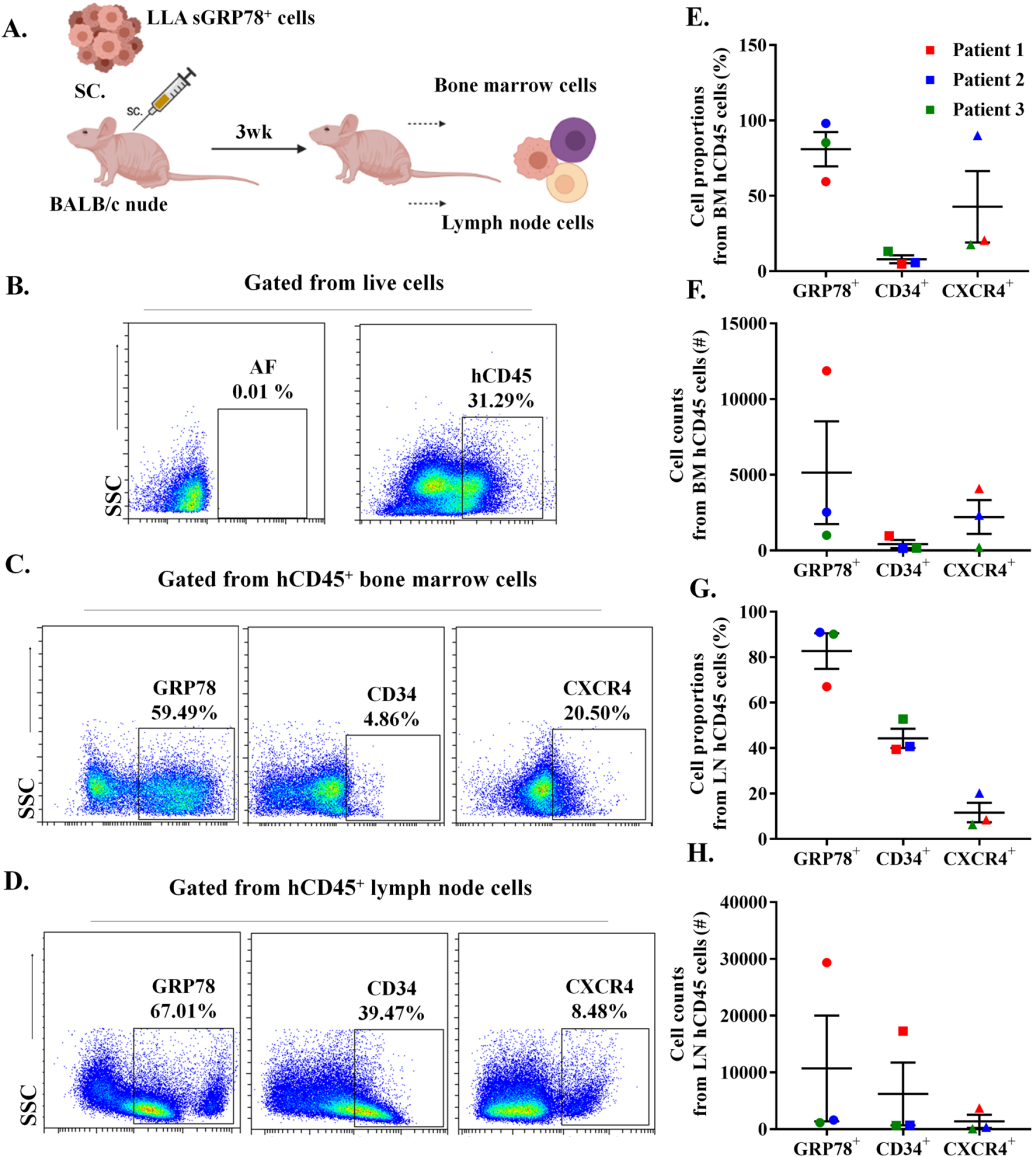
Patient-derived sGRP78^+^ leukemic cells disseminate in a xenotransplantation model. (**A**) Schematic representation of the methodology used. (**B–D**) sGRP78^+^ B-ALL human bone marrow cells were sorted from high-risk B-LLA patients and injected into a BALB/c nude immunodeficient mice. Cells from murine bone marrow (BM) and lymph node (LN) were recovered 3 weeks later and were stained with anti-human CD45 (hCD45), anti-CD34 and anti-CXCR4, representative images are depicted. (**E–H**) Percentages and absolute numbers of sGRP78^+^, CD34^+^ and CXCR4^+^ cells selected from hCD45 fraction, both in BM (**E,F**) and LN (**G,H**) are depicted. *AF* autofluorescence.

Since we observed the presence of sGRP78^+^CXCR4^+^ double positive cells in high-risk leukemia samples, both in bone marrow and peripheral blood, we wondered whether purified sGRP78^+^ cells could be ready to migrate and disseminate. Since whole leukemia cells were capable to infiltrate bone marrow and lymph nodes in nude mice (Supplementary Fig. 3); we subcutaneously injected purified High-risk patient-derived sGRP78^+^ leukemia cells (Fig. 5A) and, 3 weeks upon transplantation we detected living CD45^+^ human cells from bone marrow and lymph nodes expressing sGRP78, CXCR4 and CD34 (Fig. 5B–D). Around 80% of human recovered cells expressed sGRP78, both in bone in marrow and lymph node (Fig. 5E,G). Also, we recovered up to 30,000 human sGRP78^+^ cells in the best-case scenario (Fig. 5F,H). Compared to lymph nodes, the percentage of CXCR4^+^ cells were higher in bone marrow while the percentage of CD34^+^ cells were lower in this tissue (Fig. 5E,G). Our data suggest that sGRP78^+^ leukemia cells can migrate and lodge different tissues, and that some cells are able to maintain surface markers associated to the leukemic cell phenotype.

## Discussion

Recently it has been observed that GRP78 plays a role in survival and death processes in cancer cells^1, 27^. GRP78 is overexpressed in different types of leukemias^20, 21, 28^. Interestingly, it has been pointed out the relevance of GRP78 on the cellular surface as a hallmark of a stem-like population in cancer^25^. sGRP78 has been associated with relapse in myeloid leukemia suggesting that its presence plays a role in the maintenance of leukemia cells^7^. In solid tumor studies, sGRP78 helped to select breast and prostate cancer cells lines that are chemotherapy resistant^29^. However, it is unknown whether sGRP78 could be related to childhood leukemia at diagnosis and more importantly, if sGRP78 may be important as an indicator of High-risk leukemia. By using a multiparametric flow cytometry approach, we investigated the presence of sGRP78 in leukemic cell lines and in a set of samples derived from bone marrow of pediatric patients with B-LLA at diagnosis. We confirmed that sGRP78 is highly enriched in the Sup-B15 cell line as was observed in proteomic analysis^30^, interestingly just a small population of Reh and RS4;11 expressed sGRP78, suggesting that their molecular signatures may influence the translocation of GRP78 to the membrane cell surface. Since the Sup-B15 cell line was established from a patient with second relapse, we hypothesized that sGRP78 could be associated with High-risk leukemia.

We were able to determine that the majority of the analyzed BM samples contained a fraction with a high proportion of sGRP78^+^ cells, both in the Standard and High-risk groups, suggesting that leukemic cells relocate GRP78 to the cell surface independently of the leukemic risk group (when number of leucocytes and age are considered; see material and methods). This may reflect an actively functioning UPR response in leukemic cells, conferring aggressive properties associated to treatment resistance and relapse as has been observed in other types of cancer^31–33^. Furthermore, it may also indicate that sGRP78^+^ cells can be selected to survive once the treatment is initiated^12, 34^.

Our data suggested that the presence of sGRP78 may be intrinsically associated with early stages of leukemia. Additionally, GRP78 expression is linked to hematopoietic stem cells survival and proposed as a regulator of pluripotency and oncogenesis in other cell types^7, 25^. We decided to analyze the presence of markers for B-cell development such as CD34, CD38 and CD10 together with sGRP78. Interestingly, the fraction of sGRP78^+^ cells derived from High-risk patients co-expressed CD34, CD38 or CD10 in a significant manner compared to the fraction of sGRP78^+^ cells obtained from Standard-risk patients, suggesting that High-risk patients have higher proportions of potential primitive sGRP78^+^ leukemic cells that may represent a B-cell precursor acute lymphoblastic leukemia^35^.

Taking advantage of flow cytometry combined with high dimensional analysis, we observed a distinctive cluster of cells that are CD10^+^CD19^+^CXCR4^+^sGRP78^+^ (cluster 2) associated with pediatric High-risk leukemia at diagnosis. Also, a distinctive cluster (cluster 9) observed in Standard-risk group shared leukemic markers like CD34 and sGRP78, with low expression of CXCR4. Thus, for the first time we are showing that the presence of both sGRP78 and CXCR4 are associated with High-risk leukemia at diagnosis but not with Standard-risk leukemia.

Additionally, two clusters with elevated CD38 but low CD34 were observed in the Standard risk group (cluster 3 and 8), interestingly none of them showed marked expression of sGRP78. This finding supports previous data indicating that the subset CD38^+^CD34^-^ is associated with better prognosis in B-LLA^36^.

Also, we explored whether ALL-sGRP78 cells were also located in circulation. We showed that blood cells from leukemia patients also expressed sGRP78. Moreover, the sGRP78^+^CXCR4^+^ cell subset was enriched in the High-risk group similar to the findings observed in bone marrow cells. Thus, the presence of sGRP78^+^CXCR4^+^ leukemic blood cells may indicate that this subset could seed peripheral tissues as has been observed in solid tumors^37, 38^. On the other hand, sGRP78^+^ cells were quite absent in the control group, similarly to the phenotype observed in bone marrow, suggesting that the presence of sGRP78 in circulating cells reflects the presence of a leukemic niche in bone marrow in leukemic patients.

Since GRP78 facilitates cancer cell invasion and seeding^39^, we analyzed whether sGRP78^+^ leukemia cells could disseminate in a mouse model of xenotransplantation. We found that subcutaneously injected sGRP78^+^ cells emigrated and survived for at least 3 weeks in the transplanted mice, as we were able to detect them in bone marrow and lymph nodes. Although the percentages and absolute numbers of recovered cells from xenografts were mixed, probably because of phenotype changes of the transplanted cells or altered migratory capabilities influenced by the CXCL12/CXCR4 axis^40, 41^; the majority of living human CD45^+^ cells maintained the expression of sGRP78 and a less proportion kept the expression of CD34 and CXCR4. Although, several mouse models have been used to analyze the dissemination, homing and tumor formation capability of leukemia cells^42^, our data also support that BALB/c nude immunodeficient mice could be used to study the dissemination process of acute leukemia via subcutaneous injection.

Taken together, we propose that sGRP78 is a surface marker likely associated with stem-like and migratory phenotype of leukemia cells from pediatric patients with B-ALL at diagnosis. Thus, the evaluation of sGRP78^+^CXCR4^+^ markers along with CD10 and CD19 in leukemic pediatric samples at diagnosis could contribute to a better categorization of High and Standard-risk pediatric leukemia. Whether the presence of sGRP78^+^CXCR4^+^ leukemic cells could lead to an increased risk of relapse, which is presumed to occur in patients with myeloid leukemia that express high levels of GRP78 transcripts^7, 39^ should be further evaluated. Finally, the presence of sGRP78 in cancer cells and its absence or low expression in normal cells, makes it a potential targetable marker. There is evidence showing that GRP78 confers multidrug resistance properties to cancer cells, thus elimination of sGRP78^+^ may improve treatments for childhood leukemia.

## Supplementary Information

Supplementary Figure 1.

Supplementary Figure 2.

Supplementary Figure 3.
