# Supplementary figures and images for: Cell surface expression of GRP78 and CXCR4 is associated with childhood high-risk acute lymphoblastic leukemia at diagnostics
Source: Sci Rep. 2022 Feb 11;12:2322. doi: 10.1038/s41598-022-05857-w (PMC8837614; doi:10.1038/s41598-022-05857-w)

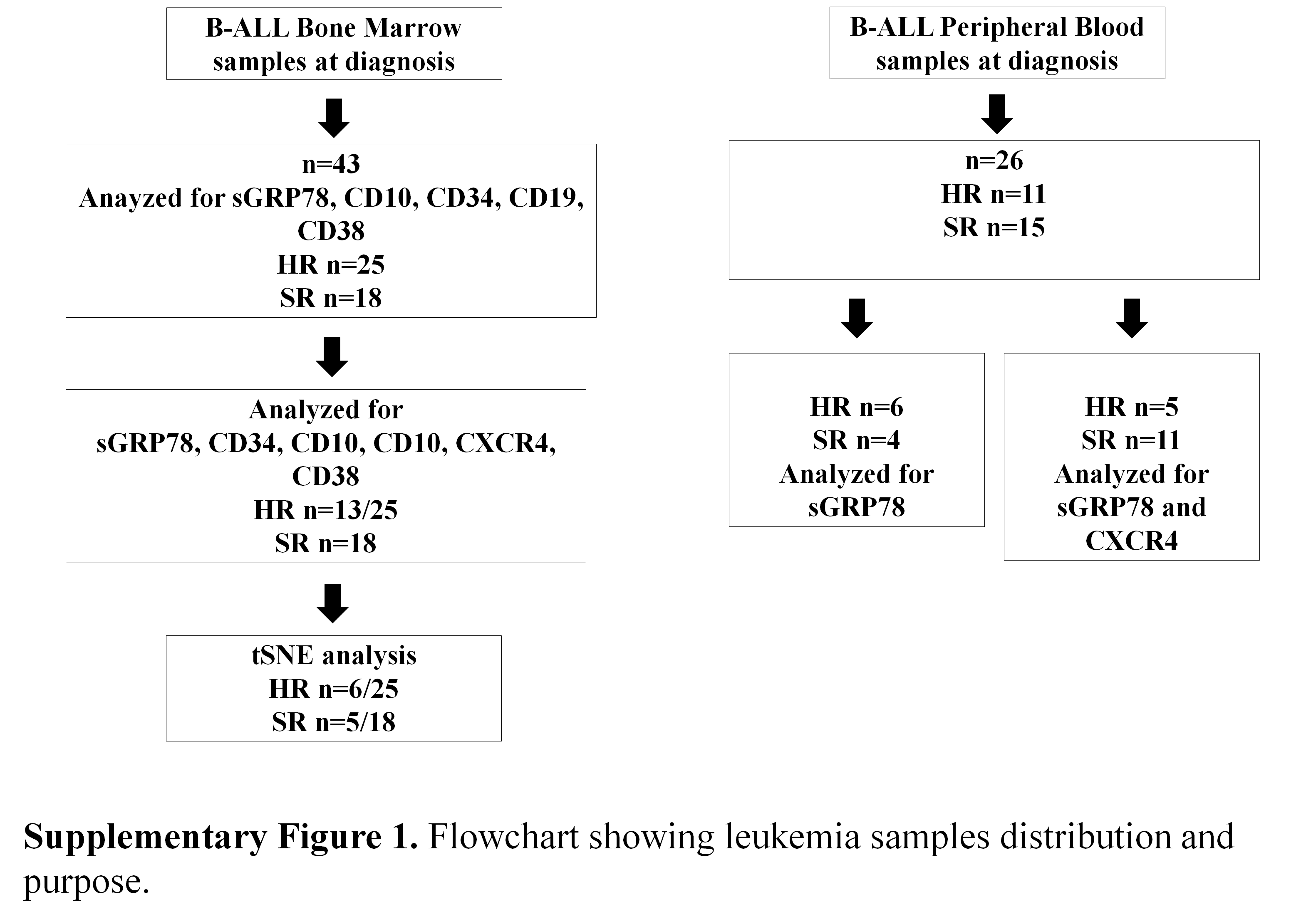

Supplement: Supplementary file 1 — Supplementary Figure 1. [file 41598_2022_5857_MOESM1_ESM.tif]

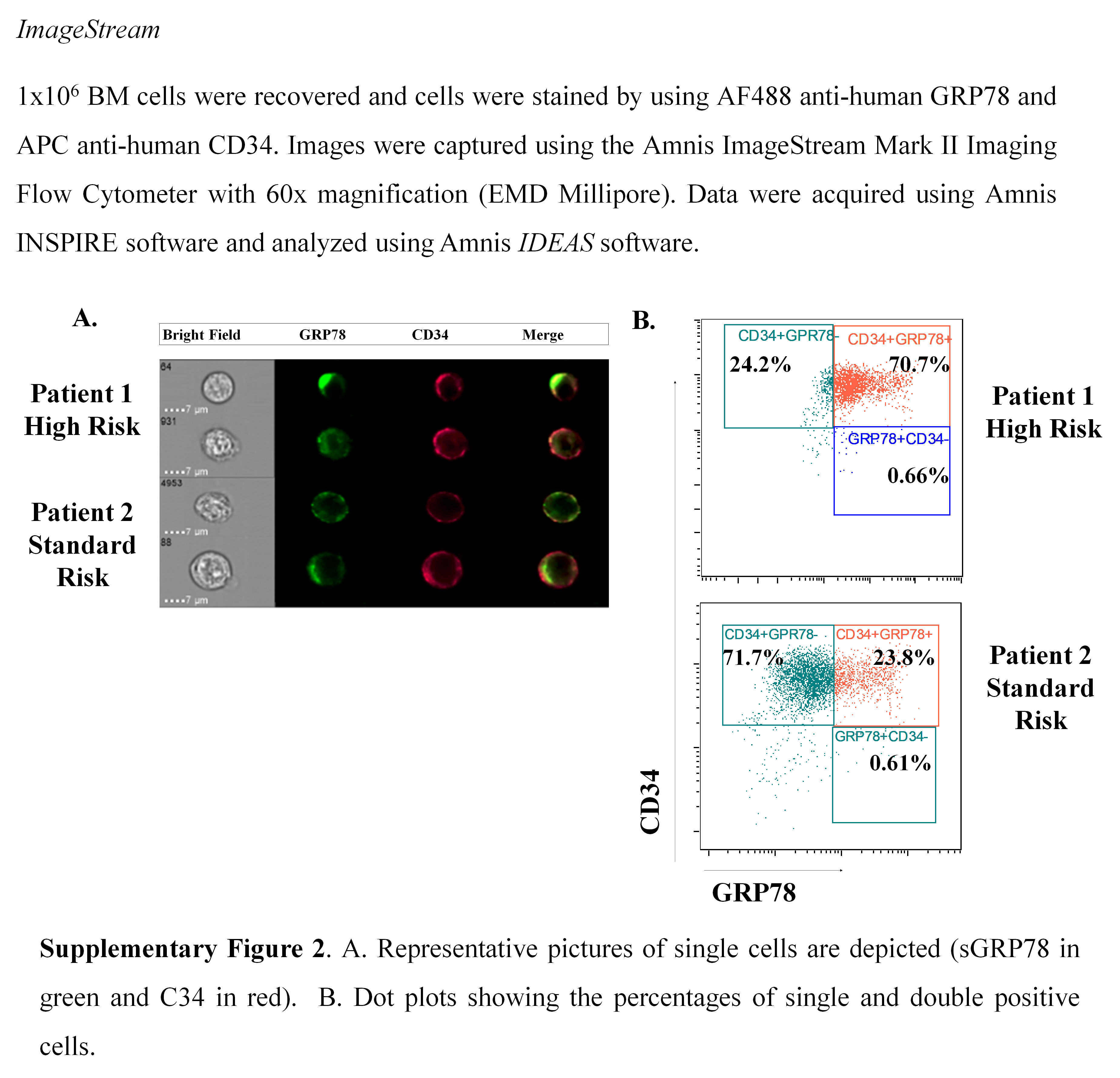

Supplement: Supplementary file 2 — Supplementary Figure 2. [file 41598_2022_5857_MOESM2_ESM.tif]

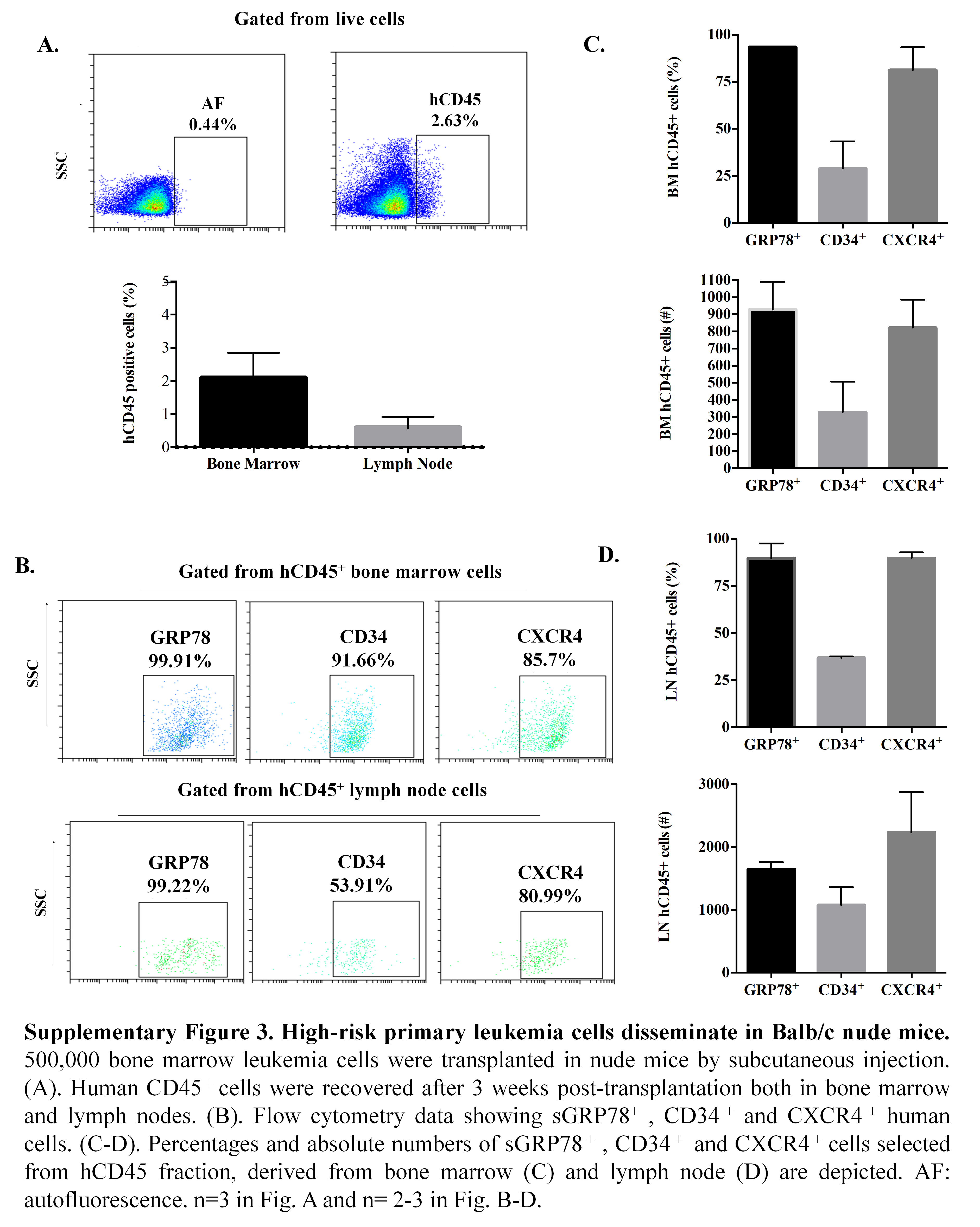

Supplement: Supplementary file 3 — Supplementary Figure 3. [file 41598_2022_5857_MOESM3_ESM.tif]
